# Factors Affecting the Time to Postoperative Recovery of Consciousness in Patients Undergoing Cardiac Surgery With Cardiopulmonary Bypass: A Retrospective Study

**DOI:** 10.7759/cureus.80227

**Published:** 2025-03-07

**Authors:** Mikiko Tomino, Ryoji Maeda, Kazuharu Harada, Shinya Motohashi, Toru Goyagi

**Affiliations:** 1 Anesthesiology, Tokyo Medical University Hachioji Medical Center, Tokyo, JPN; 2 Anesthesiology, Tokyo Medical University, Tokyo, JPN; 3 Health Data Science, Tokyo Medical University, Tokyo, JPN; 4 Cardiovascular Surgery, Tokyo Medical University Hachioji Medical Center, Tokyo, JPN

**Keywords:** anesthesia recovery period, anesthetics, cardiac surgical procedures, cardiopulmonary bypass, consciousness

## Abstract

Introduction: The causes of delayed recovery of consciousness from anesthesia after cardiac surgery with cardiopulmonary bypass are diverse and often related to multiple factors. This study investigated the factors affecting the time to recovery of consciousness in patients undergoing cardiac surgery with cardiopulmonary bypass at our hospital.

Methods: The retrospective observational study included 113 patients aged ≥20 years who underwent cardiac surgery with cardiopulmonary bypass at Tokyo Medical University Hachioji Medical Center between January 2019 and December 2022. Patients who underwent cardiovascular surgery with selective cerebral perfusion, patients who underwent cardiac surgery without cardiopulmonary bypass, patients with preoperative impaired consciousness, patients who used sedatives preoperatively, patients with postoperative cerebrovascular complications, patients who refused to consent to participate in the study, and patients whose information necessary for the study was not recorded were excluded. Preoperative and intraoperative variables that could affect the time to recovery of consciousness were selected for statistical analysis. Univariate and multivariate linear regression analyses were performed to assess the association of these factors with time to recovery of consciousness.

Results: Univariate linear regression analysis revealed that albumin, estimated glomerular filtration rate (eGFR), and bicaudate ratio (BCR) were significantly associated with the time to recovery of consciousness. In the multivariate linear regression analysis, eGFR and BCR had significant associations. The regression coefficients were negative for eGFR and positive for BCR. The reduced model obtained through the backward step-down methods showed an improved fit and included these significant variables and other factors.

Conclusions: Preoperative renal dysfunction and brain atrophy were identified as factors affecting the postoperative time to recovery of consciousness after cardiac surgery with cardiopulmonary bypass. Additionally, cardiopulmonary bypass time and hypoalbuminemia suggested that they were related to the time to recovery of consciousness. For patients with renal dysfunction, brain atrophy, or hypoalbuminemia, adjusting the dose and timing of anesthetic agent administration during induction and surgery may help reduce the time to recovery of consciousness.

## Introduction

Factors that affect the time to recovery of consciousness (ROC) from anesthesia include residual anesthetic, cerebrovascular, metabolic, and ventilatory disturbances and abnormal body temperature. In cardiac surgery with cardiopulmonary bypass (CPB), procedure-related factors such as hypothermia, hemorrhagic stroke due to anticoagulation, cerebral ischemia due to microemboli, and altered cerebral blood flow can cause delayed postoperative ROC [1]. However, identifying all relevant contributors to delayed ROC is difficult due to the multifactorial influences involved.

Cardiac surgery was previously managed using high-dose opioid-based anesthesia and long-term postoperative ventilatory management [2]. However, fast-track cardiac anesthesia (FTCA), which emphasizes early or immediate extubation, has been widely adopted. FTCA has not been associated with increased mortality or major complications compared with conventional anesthesia management [3]. While it has not significantly shortened hospital stays, it has contributed to healthcare cost reduction by decreasing extubation time and intensive care unit (ICU) length of stay [3]. A recent systematic review reported that the factors contributing to delayed extubation included pre-existing heart failure, renal function, CPB time, and aortic cross-clamp time [4].

Even in cardiac surgery performed with anesthetic methods conducted to the same standard, patients who do not recover from anesthesia for long periods are encountered. Chen et al. defined delayed ROC as not recovering 12 hours after completion of anesthetic administration and reported that age, CPB time, duration of surgery, high blood urea nitrogen (BUN) levels, and presence of red blood cell transfusion increased the risk of delayed ROC [5]. Furthermore, age, CPB time, sex, preoperative BUN level, and body mass index (BMI) are related to the time to ROC after cardiac surgery [6,7]. To determine whether these factors are relevant in our institution, we retrospectively studied factors affecting the postoperative time to ROC in patients undergoing cardiac surgery with CPB. The aim of this study is to confirm existing findings and explore additional variables specific to our institution. Specifically, we aim to identify the factors that affect ROC and, by clarifying these factors, contribute to shortening the ROC through improved postoperative management and preoperative intervention.

This article was presented previously as an oral presentation at the 194th Tokyo Medical University Medical Association General Meeting on November 2, 2024.

## Materials and methods

Ethical approval

This single-center, retrospective, observational study was conducted at Tokyo Medical University Hachioji Medical Center. The Medical Ethics Committee of Tokyo Medical University approved this study on June 9, 2023 (T2023-0048) and waived the requirement for written informed consent. However, the study was publicly announced on the website of the Tokyo Medical University Hachioji Medical Center, and the opportunity to opt out was provided. Patients were notified via the hospital website, but not individually, and no patients opted out. Chart reviews were accessed for research purposes from June 10, 2023, to November 30, 2023. All data obtained from electronic hospital records were stripped of personal identifiers, such as name, date of birth, and address, and replaced with a research registration number to ensure anonymity. As such, the authors did not have access to information that could identify individual participants after data collection.

Inclusion and exclusion criteria

**Figure 1:**
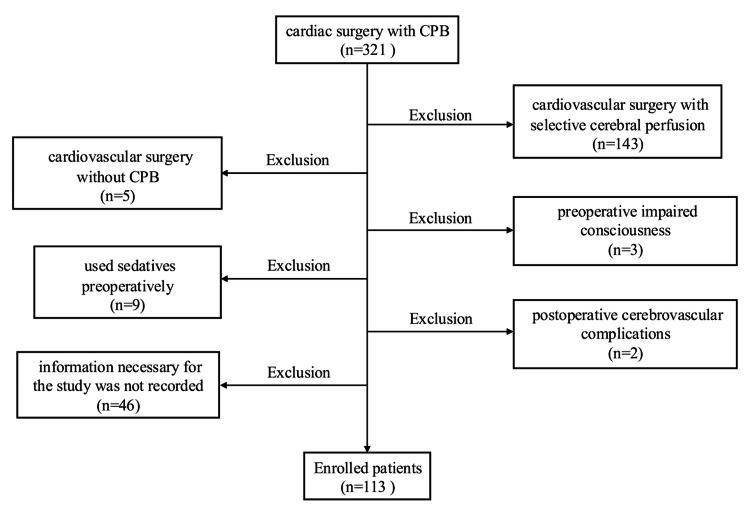
Flow diagram of research participants. CPB: cardiopulmonary bypass.

Of all patients aged ≥20 years who underwent cardiac surgery with CPB between January 2019 and December 2022, those meeting the exclusion criteria were excluded, resulting in a final study population of 113 patients (Figure 1). The exclusion criteria were as follows: patients who underwent cardiovascular surgery with selective cerebral perfusion, patients who underwent cardiac surgery without CPB, patients with preoperative impaired consciousness, patients who used sedatives preoperatively, patients with postoperative cerebrovascular complications, patients who requested not to participate in the study, and patients whose information necessary for the study was not recorded.

Anesthesia and postoperative management

After entering the operating room, the patient was given local anesthesia at the site where the arterial line was inserted, and the arterial line was inserted. Before induction of anesthesia, regional oxygen saturation (rSO_2_) was continuously measured at the forehead area using the INVOS^TM^-5100C oximeter (Medtronic, Dublin, Ireland) for near-infrared spectroscopy measurements. A baseline measurement was taken before the induction of anesthesia in all patients. A bispectral index (BIS) monitor, five-point electrocardiogram, and peripheral oxygen saturation (SpO_2_) were attached, and anesthesia was induced. After the induction of anesthesia, transesophageal echocardiography and thermometry (bladder and rectal temperatures) were performed.

Anesthesia was induced with midazolam, remifentanil, fentanyl, and rocuronium in all patients, and maintenance was performed with inhalation anesthesia using sevoflurane or total intravenous anesthesia using propofol with target-controlled infusion (TCI). There was no special sedation protocol during anesthesia, and sedation was adjusted so that the BIS value was between 40 and 60 for all patients. All patients received propofol at a dose of 2 μg/mL effector site concentration TCI during CPB. In all cases, the arterial cannula for blood supply was inserted into the ascending aorta. The venous cannula was inserted as a single vessel from the right atrium to the inferior vena cava in cases where the right atrium was not incised (e.g., coronary artery bypass graft (CABG) and aortic valve replacement (AVR)). In cases where the right atrium was incised, two venous cannulas were used: one in the superior vena cava and one in the inferior vena cava. Using a centrifugal pump, the perfusion index was maintained at a steady flow rate of 2.3 mL/min/m^2^, regardless of age. The target hematocrit level was between 18% and 20%, and the target perfusion pressure was 60-70 mmHg using blood transfusions and vasoactive drugs. Body temperature was maintained at a mild hypothermia of between 32°C and 34°C in cases of aortic cross-clamp, such as valvular surgery, and not lower than 35°C in pulsatile CABG. In the case of CABG alone, on-pump beating was used, and aortic cross-clamping was not used. In addition to continuous blood monitoring, blood gas analysis was performed every 30 minutes, and activated clotting time was performed every 60 minutes and corrected accordingly.

After the surgery was completed, the patients were admitted to the ICU under controlled ventilation. After that, the patients were sedated with 2% propofol at 1 mL/h, and the ventilator was set such that the partial pressure of oxygen (PaO_2_) was >100 mmHg (fraction of inspired oxygen (FiO2) = 0.4) and partial pressure of carbon dioxide (PaCO_2_) was 35-40 mmHg. After confirming ROC by eye-opening, the absence of tetraplegia, and the ability to follow commands, a continuous infusion of dexmedetomidine was started. After the start of dexmedetomidine administration, the ventilator was disconnected while the patient was receiving dexmedetomidine when the patient's circulatory and respiratory conditions stabilized.

Study design

Patient measurements and laboratory data were extracted from electronic medical, anesthesia, and cardiopulmonary records. Preoperative data included serum concentrations of aspartate aminotransferase (AST), alanine aminotransferase (ALT), estimated glomerular filtration rate (eGFR), albumin, left ventricular ejection fraction (LVEF), presence or absence of coexisting pulmonary disease, hypertension, and diabetes mellitus, and preoperative intra-aortic balloon pump (IABP) insertion. Hepatic dysfunction was defined as when both AST and ALT values exceeded the reference values (AST > 33 U/L; ALT > 43 U/L), and renal dysfunction was defined using eGFR values as normal (eGFR ≥ 90 mL/min/1.73 m2), mild (90 > eGFR ≥ 60 mL/min/1.73 m^2^), moderate (60 > eGFR ≥ 30 mL/min/1.73 m^2^), severe (30 > eGFR ≥ 15 mL/min/1.73 m^2^), and end-stage renal failure (eGFR <15 mL/min/1.73 m^2^). Liver function tests and eGFR measurements were performed immediately before surgery. Cardiac function was assessed using LVEF and classified into LVEF ≤40%, 41-49%, and ≥50% based on the classification of heart failure [8]. Anesthesia records, anesthesia time, operation time, CPB time, blood transfusion, type and dose of anesthetics, intraoperative cardiac pacing, emergency surgery, and body fluid balance (in-out balance at the end of surgery) were extracted. From the postoperative data, body temperature at ICU admission and ROC time were extracted. rSO_2_ values were compared before anesthesia induction, during CPB (lowest value), and at the end of surgery. According to the “Guidelines for the use of cerebral oximetry by near-Infrared spectroscopy in cardiovascular anesthesia,” a 20% decrease from the baseline value during cardiac surgery may reduce the risk of brain damage, so we defined the threshold for a decrease in rSO_2_ as 20% [9]. The degree of brain atrophy was evaluated one-dimensionally by calculating the bicaudate ratio (BCR) using preoperative magnetic resonance imaging (MRI) or computed tomography (CT) of the brain, which were performed on all patients prior to surgery for a detailed examination of cerebrovascular lesions. The BCR was measured using images of the caudate nucleus level and was calculated by dividing the width of the caudate nucleus by the white matter width at the same level as the caudate nucleus. The values were measured manually by two anesthesiologists, and the average value was calculated to ensure objectivity, but inter-rater reliability analysis was not performed. Factors that might affect the ROC time, including age, sex, BMI, liver function, renal function, degree of brain atrophy, LVEF, pulmonary disease, hypertension, diabetes mellitus, and preoperative IABP, were examined. Anesthetic factors, including the total intraoperative doses of fentanyl and midazolam, were examined. Surgical factors were examined, including anesthesia time, CPB time, intraoperative cardiac pacing, blood transfusion, body fluid balance, temperature at ICU entry, and rSO_2 _values.

Statistical analysis

Data are summarized as median (q1-q3). Histograms and Q-Q plots were used to confirm the distribution of ROC times and to examine whether log transformation was necessary. Factors that might affect the ROC time were examined using univariate and multivariate linear regression analyses. For multivariate linear regression analysis, we created a full model in which all factors were included and a reduced model in which unnecessary variables were removed using the backward step-down method. The exclusion criterion for variable elimination was P > 0.5 based on the F-test for structural changes, which is an approximation of the full model by deleting non-relevant variables [10]. The regression coefficients and their P-values were presented. Sex was assigned 0 for males and 1 for females, and liver function was assigned 0 for normal and 1 for abnormal; no significant change in rSO_2_ was assigned 0, and a significant decrease was assigned 1. LVEF was assigned to three categories (≤40%, 41-49%, and ≥50%); surgery was assigned 0 for elective surgery and 1 for emergency surgery; pulmonary disease, hypertension, diabetes mellitus, blood transfusion, IABP, and pacing were each assigned 0 for none and 1 for yes. The significance level was set at P < 0.05. Data were analyzed using SPSS Statistics for Macintosh, version 29.0 (IBM Corp., Armonk, NY).

## Results

The sex, age, BMI, and preoperative complications of the 113 eligible patients are shown in Table 1. All tests were measured immediately before surgery. Age adjustment was not performed for BCR. None of the patients with cerebrovascular disease noted on preoperative MRI or CT of the head were symptomatic. The surgical procedures for the eligible patients are presented in Table 2. The intraoperative and postoperative patient data are shown in Table 3. No major complications were observed in any of the patients. Blood transfusions refer only to red blood cell transfusions. The decrease in rSO_2_ was not a clinically significant decrease, and no association with postoperative outcomes was observed. No new cerebrovascular accidents or deaths occurred in any of the patients.

**Table 1:** Basic characteristics of included patients. Data are presented as median or n (%). BMI: body mass index; eGFR: estimated glomerular filtration rate; LVEF: left ventricular ejection fraction; IABP: intra-aortic balloon pumping; BCR: bicaudate ratio; Q1-Q3: 1st and 3rd quartile.

Variables		Median (Q1-Q3)	n (%)
Sex (male)			81 (72)
Age, years		73 (67-78)	
BMI, kg/m^2^		22 (19.9-24.5)	
Impairment of liver function			8 (7)
Albumin, g/dL		3.6 (1.7-4.6)	
eGFR, mL/min/1.73 m^2^		54.2 (28.7-64.6)	
Renal dysfunction	Normal		1 (1)
	Mild		40 (35)
	Moderate		42 (37)
	Severe		7 (6)
	End-stage renal failure		23 (20)
LVEF	≤41%		17 (15)
	41–49%		21 (19)
	≥50%		75 (66)
Pulmonary disease			10 (9)
Hypertension			72 (64)
Diabetes mellitus			41 (36)
Preoperative IABP			7 (6)
Cerebrovascular disease	Cerebral infarction		45 (40)
	Cerebral hemorrhage		6 (5)
	Both		4 (4)
BCR, %		14.1 (12.4-16.1)	

**Table 2:** Surgical procedures of included patients. Data are presented as median or n (%). CABG: coronary artery bypass graft.

Surgery	n (%)
CABG	37 (33%)
Valvular surgery	60 (53%)
CABG + valvular surgery	14 (12%)
CABG + left ventriculoplasty	1 (1%)
Left atrial myxoma	1 (1%)

**Table 3:** Intraoperative and postoperative information of included patients. Data are presented as median or n (%). CPB: cardiopulmonary bypass; rSO_2_: regional saturation of oxygen; BT: body temperature; ICU: intensive care unit; ROC: recovery of consciousness; Q1-Q3: 1st and 3rd quartile.

Variables		Median (Q1-Q3)	n (%)
Emergency surgery			6 (5)
Anesthesia time, minutes		394 (325-470)	
Operation time, minutes		297 (228-359)	
CPB time, minutes		122 (90-165)	
Total dose of fentanyl, μg/kg		8.7 (7.6-10.3)	
Total dose of midazolam, mg/kg		0.15 (0.12-0.18)	
Transfusion			91 (74)
Body fluid balance, mL		2962 (2384-4748)	
Requirement for intraoperative cardiac pacing			21 (19)
rSO_2_ decrease (CPB)			36 (32)
	Bilateral sensors		25 (22)
	Unilateral sensors		11 (10)
rSO_2_ decrease (leaving OR)			15 (13)
	Bilateral sensors		9 (8)
	Unilateral sensors		6 (5)
BT (ICU), ℃		35.7 (35.3-36.0)	
ROC time, minutes		195 (130-300)	

**Figure 2:**
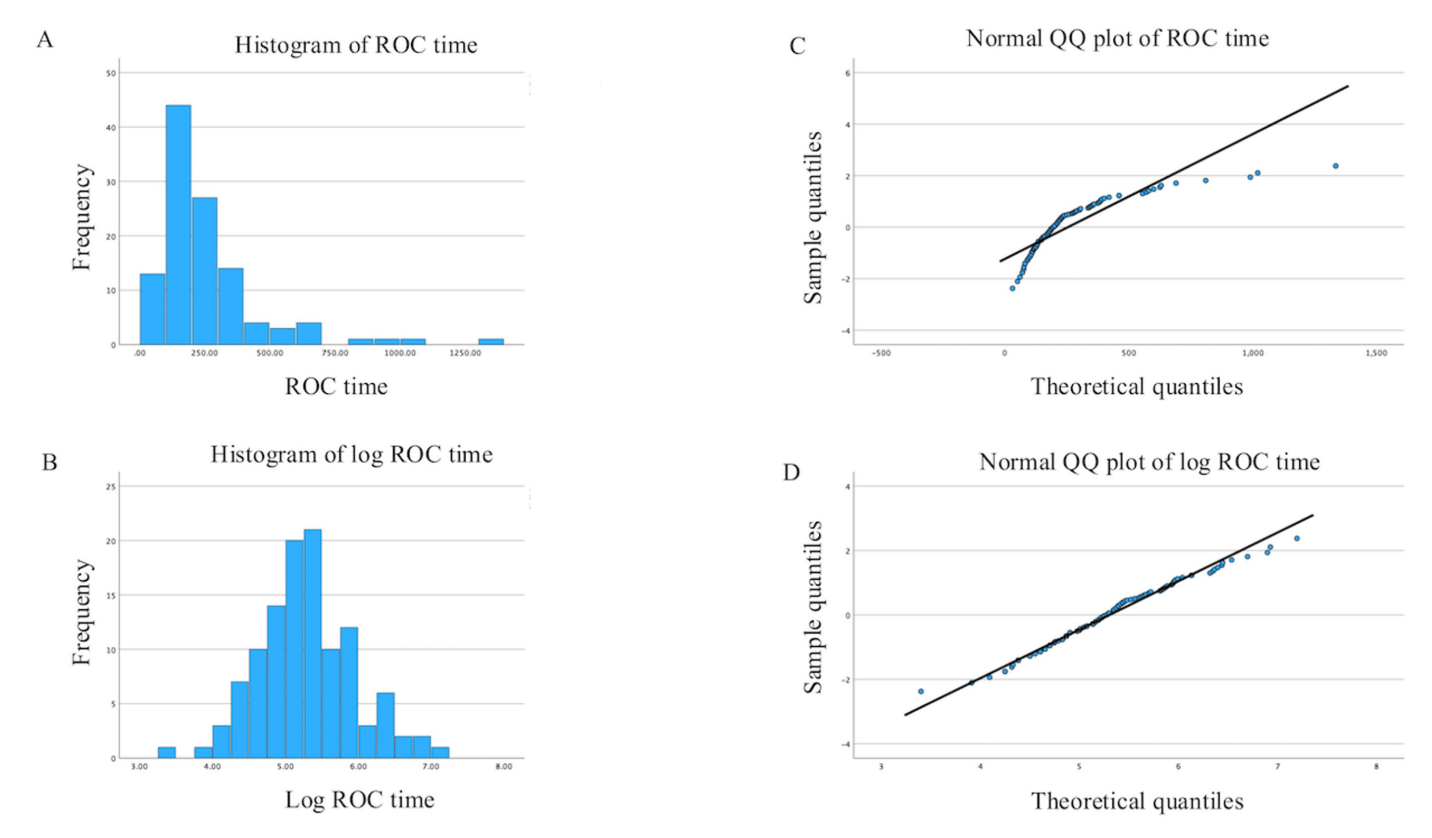
Histogram and normal QQ plot. (A) Histogram of ROC time. The distribution of ROC time was positively skewed. (B) Histogram of log ROC time. The histogram is more symmetrical than before the transformation. (C) Normal QQ plot of ROC time. The data points deviate from the theoretical quantiles of a normal distribution (black line). (D) Normal QQ plot of log ROC time. The data points align along the theoretical quantiles. QQ plot: quantile-quantile plot; ROC: recovery of consciousness.

Because the distribution of the ROC time was positively skewed, a log transformation was performed (Figure 2). The results of the univariate and multivariate linear regression analyses of the factors affecting the ROC time are shown in Table 4. In the regression analyses, one patient with missing albumin was excluded. In the univariate regression analysis, many factors were significantly associated with ROC time, particularly albumin level, eGFR, and BCR, which showed a small P-value (P < 0.001). In the multivariate regression analysis using the full model, eGFR and BCR were significantly associated with the ROC time (P < 0.05). Although the significance level was not met, albumin level and CPB time were associated with ROC time with a relatively small P-value (P = 0.086 and 0.093, respectively). The reduced model included sex, age, BMI, eGFR, LVEF, total intraoperative doses of fentanyl and midazolam, BCR, albumin level, CPB time, and emergency surgery. The adjusted R-squared values for the full and reduced models were 0.431 and 0.491, respectively, indicating that the model fit was improved by removing unnecessary variables. The models were validated post-hoc from several perspectives. The maximum absolute correlation between the explanatory variables was 0.568, and the variance inflation factor (VIF) was at most 3.036 (full model), indicating that multicollinearity was not an issue. The regression residuals followed a roughly normal distribution (P = 0.762, Shapiro-Wilk), and by examining the scatterplot of predicted values versus residuals, we confirmed that the assumptions of linearity and homoscedasticity were valid. Additionally, as a sensitivity analysis for the single missing data point, we performed the same analysis using a dataset in which the albumin value for that case was imputed with the mean, and the results for both the full model and the reduced model were nearly identical. As fentanyl and midazolam were administered in small doses, it is thought that they did not affect the ROC time. There was a great deal of variation in the data regarding body fluid balance, and this was thought to be due to the influence of abnormal values.

**Table 4:** Univariate and multivariate analysis of factors potentially influencing recovery of consciousness time after elective cardiac surgery. Coef.: regression coefficient; CI: confidence interval; BMI: body mass index; Alb: albumin; eGFR: estimated glomerular filtration rate; LVEF: left ventricular ejection fraction; BCR: bicaudate ratio; CPB: cardiopulmonary bypass; BT: body temperature; ICU: intensive care unit; rSO_2_: regional saturation of oxygen; OR: operating room.

	Univariable				Multivariable				
					Full model				Reduced model
	Coef.	95% CI		P-value	Coef.	95% CI		P-value	Coef.
		Lower limit	Upper limit			Lower limit	Upper limit		
Intercept					4.653	-2.35	11.657	0.19	4.614
Sex	-0.233	-0.505	0.039	0.093	-0.061	-0.427	0.304	0.739	-0.146
Age	0.014	0.002	0.026	0.022	0.006	-0.005	0.018	0.294	0.006
BMI	-0.036	-0.068	-0.003	0.031	-0.015	-0.051	0.022	0.424	-0.014
Impairment of liver function	-0.156	-0.639	0.327	0.524	0.118	-0.282	0.518	0.558	-
Alb	-0.464	-0.65	-0.277	<0.001	-0.176	-0.378	0.025	0.086	-0.179
eGFR	-0.014	-0.018	-0.01	<0.001	-0.01	-0.015	-0.005	<0.001	-0.009
LVEF ≥50%	(Ref)	-	-	-	(Ref)	-	-	-	-
41-49%	0.231	-0.085	0.547	0.15	0.117	-0.161	0.395	0.406	0.11
≤40%	0.033	-0.315	0.38	0.852	-0.145	-0.476	0.186	0.385	-0.117
Pulmonary disease	0.172	-0.264	0.608	0.436	-0.018	-0.37	0.334	0.918	-
Hypertension	0.153	-0.104	0.409	0.241	0.061	-0.178	0.301	0.612	-
Diabetes mellitus	0.297	0.045	0.549	0.021	-0.054	-0.307	0.2	0.675	-
Preoperative IABP	0.12	-0.395	0.634	0.646	0.082	-0.432	0.595	0.753	-
BCR	0.129	0.091	0.166	<0.001	0.076	0.031	0.121	0.001	0.074
Emergency surgery	0.074	-0.48	0.627	0.793	-0.341	-0.854	0.172	0.189	-0.286
Anesthesia time	0.002	0.001	0.003	0.003	0	-0.001	0.002	0.884	-
Total dose of fentanyl	0.01	-0.049	0.068	0.744	0.015	-0.05	0.08	0.639	0.022
Total dose of midazolam	0.849	-2.077	3.775	0.566	1.505	-1.413	4.423	0.308	1.228
CPB time	0.003	0.001	0.005	0.005	0.002	0	0.004	0.093	0.002
BT (ICU)	-0.076	-0.278	0.125	0.454	-0.002	-0.187	0.183	0.981	-
rSO_2_ decrease (CPB)	0.017	-0.249	0.284	0.898	-0.025	-0.273	0.222	0.841	-
rSO_2_ decrease (leaving OR)	0.162	-0.203	0.527	0.38	0.013	-0.324	0.35	0.94	-
Intraoperative cardiac pacing	-0.144	-0.462	0.174	0.371	-0.075	-0.341	0.191	0.579	-
Body fluid balance	0	0	0	0.003	1.447	0	0	0.719	-
Transfusion	0.487	0.187	0.787	0.002	-0.09	-0.437	0.257	0.606	-
	Adjusted R^2^				0.431				0.491
	Model F-test				<0.001				<0.001

## Discussion

This study examined the factors that influenced the postoperative ROC time after cardiac surgery with CPB. Although multiple factors may affect ROC time, previous studies have identified several key contributors to delayed ROC and extubation [4-7]. In addition to these previously reported factors, our study included anesthesia time, total intraoperative amount of fentanyl and midazolam, and fluid balance as study items. We also assessed brain function by examining brain atrophy and intraoperative rSO_2 _changes. The results showed that preoperative renal function and brain atrophy significantly affected the ROC time. The results of the univariate regression and the reduced model for CPB time and albumin serum concentrations also suggested that they were related to the ROC time to some extent. In contrast, sex, age, BMI, emergency surgery, and the total intraoperative amount of fentanyl and midazolam did not affect the ROC time. Furthermore, while some factors associated with delayed extubation were examined, most showed little relationship with the ROC, except for renal function and CPB time.

Renal function has been previously reported as a factor affecting ROC time, but brain atrophy has never been examined, and this study revealed a new associated factor. Long-term ventilatory management causes respiratory complications, such as atelectasis and pneumonia, and the importance of early extubation has attracted attention [4,11]. A delayed ROC can delay the detection and intervention of postoperative neurological complications, making it important to investigate its associated factors. Preoperative brain atrophy affects the ROC time; in this study, the BCR, a one-dimensional measure of brain atrophy index, was used, allowing evaluation using a simple method. Preoperative cognitive function was not assessed; therefore, this association remains an issue for future studies.

Renal dysfunction significantly impacts drug metabolism and excretion. Midazolam, a commonly used anesthetic, is primarily excreted by the kidneys, and its accumulation in patients with renal failure may contribute to prolonged impaired consciousness. Additionally, propofol is known to interact with midazolam by reducing clearance and enhancing its pharmacological effects [12,13]. In addition, propofol is believed to replace albumin-bound midazolam and increase the free midazolam levels, which may be a contributing factor [14]. Although midazolam dosage was not directly associated with ROC time in our study, its effects may be more pronounced in cases of renal dysfunction. In addition, the use of short-acting remimazolam or propofol instead of midazolam may reduce the impact on the ROC time.

Serum albumin levels were negatively correlated with ROC time, suggesting that hypoalbuminemia may enhance the effects of protein-bound anesthetics, leading to prolonged recovery. Anesthetics such as propofol, midazolam, and fentanyl are highly protein-bound, and hypoalbuminemia alone may also have an effect. Patients with renal dysfunction, particularly those on dialysis, often have lower albumin levels, which could contribute to extended ROC time.

Brain atrophy progresses with age and is accelerated by conditions such as hypertension, diabetes mellitus, and renal dysfunction [15,16]. Patients undergoing chronic dialysis experience more rapid brain atrophy, likely due to cerebral ischemia and hypotension [17-19]. This suggests that renal dysfunction may indirectly contribute to delayed ROC through its association with brain atrophy. However, in this study, it was not possible to compare the magnitude of the effect of brain atrophy with renal dysfunction or cardiopulmonary bypass time. BCR and cognitive decline have been reported to be closely related in studies of patients with multiple sclerosis [20]. Cognitive impairment may enhance the effects of anesthetics because dementia-related changes in neurotransmitter function can potentiate anesthetic mechanisms [21].

CPB is known to cause delayed ROC, with cerebrovascular complications such as stroke being common concerns. However, since patients with postoperative cerebrovascular complications were excluded from this study, other mechanisms must be considered. Systemic inflammatory responses triggered by CPB due to blood contact with the circuit, ischemia-reperfusion injury, surgical trauma, and endotoxin release lead to cytokine production, which can induce cerebral vasoconstriction and ischemic cerebral edema [22-28]. In this study, inflammatory markers were not measured. Previous studies have reported that MRI scans performed shortly after cardiac surgery with CPB show significant cerebral edema, which improves over time [29]. In contrast, off-pump CABG does not result in cerebral edema [30]. These findings suggest that CPB may temporarily contribute to postoperative cerebral edema, and the longer the CPB duration, the greater the inflammatory response, potentially affecting ROC time. In addition, there were cases in this study where aortic cross-clamping was performed and cases where it was not. Although there were no cases where aortic cross-clamping was performed and a decrease in rSO_2 _was observed, aortic cross-clamping may affect ROC time, so we would like to make this a topic for future research.

Limitations

This study has some limitations. First, it was a retrospective, single-center study with a small sample size, which limited the ability to reliably estimate regression coefficients in the full model. To address this, increasing the sample size would be ideal. Alternatively, reducing the number of analyzed factors based on clinical relevance before data analysis could have improved model stability. However, since one of the study’s objectives was to explore potential factors, we chose to include all relevant variables.

Second, we did not measure blood concentrations of anesthetic agents or monitor burst suppression using electroencephalography, which may have influenced our findings. However, the impact on the results was mitigated by BIS monitoring.

Third, the limited number of cases prevented the standardization of body temperature management during CPB and surgical procedures. This was due to the fact that the target body temperature during CPB differed depending on the surgical procedure. However, there was no variation between patients, as the body temperature was maintained at around 36℃ before and after CPB. Similarly, variations in cardiac and renal function among patients could not be fully controlled. Additionally, while a history of cerebrovascular disease - whether symptomatic or not - may have influenced ROC, excluding all such cases would have significantly reduced the study population, making the analysis impractical. Therefore, asymptomatic cases were included. As the number of asymptomatic cases was small, we did not perform subgroup analysis.

Fourth, since this study was conducted exclusively on Japanese individuals, the average BMI of the study population was lower than that of other populations, potentially limiting the generalizability of our findings.

Given these limitations, future studies with larger, more diverse cohorts and standardized methodologies are needed to enhance the robustness and applicability of the results.

## Conclusions

Factors affecting the postoperative ROC time after cardiac surgery with CPB included preoperative renal dysfunction, cerebral atrophy, and low blood albumin levels in univariate linear regression analysis. In addition, multivariate linear regression analysis included preoperative renal dysfunction and cerebral atrophy and suggested an association between CPB time and low blood albumin levels. These factors lead to residual or prolonged effects of anesthetics, suggesting the possibility of reducing the ROC time by adjusting the dose and timing of anesthetic administration. It was speculated that ROC time was particularly susceptible to the effects of midazolam. In future studies, we would like to conduct a prospective study using the incremental-dose method of anesthetic administration based on renal function and albumin levels, as well as a subgroup analysis of dialysis patients.
